# Alien Insects in Italy: Comparing Patterns from the Regional to European Level

**DOI:** 10.1673/031.013.7301

**Published:** 2013-07-30

**Authors:** Alberto F. Inghilesi, Giuseppe Mazza, Rita Cervo, Francesca Gherardi, Paolo Sposimo, Elena Tricarico, Marzio Zapparoli

**Affiliations:** 1 Dipartimento di Biologia, Università degli Studi di Firenze, Via Romana 17, 50125 Firenze, Italy; 2 NEMO, Nature and Environment Management Operators s.r.l., Piazza M. D'Azeglio 11, 50121 Firenze, Italy; 3 Dipartimento per la Innovazione nei Sistemi Biologici, Agroalimentari e Forestali, Università degli Studi della Tuscia, Via San Camillo De Lellis s.n.c., 01100 Viterbo, Italy

**Keywords:** alien species, allodiversity, checklist, Europe, Tuscany

## Abstract

The introduction of species outside their native range contributes to the loss of biodiversity, alters the structure and functioning of ecosystems, and damages economy and human health. Insects are one of the taxa with the highest frequency of introduction due to their high diversity, biological properties, and close association with human activities. Here, the allodiversity of Italian entomofauna was analyzed, with a focus on Tuscany (Central Italy). A list of alien insects in Tuscany is included. The status of the alien entomofauna in Italy was updated. The number of alien insects amounts to 122 in Tuscany and 923 in Italy. An introduction rate of 98 species per decade was estimated in Italy. In Tuscany, alien insects belong to 10 orders, mostly Coleoptera (38%), Hemiptera (Sternorrhyncha and Auchenorrhyncha) (23%), and Hymenoptera (13%). They have been most often introduced through vegetable items (ornamental plants or crops). Most species come from the Nearctic region (26%) and are both phytophagous (63%) and amphigonic (80%). Differences and similarities in introduction patterns and in insect abundances across orders among regional, national, and European scales, also considering worldwide abundances, are discussed. Finally, a paucity of information regarding the negative impacts of many species, except for economic pests, phytosanitary threats, and vectors of disease, is underlined. A deeper understanding of the alien insects' ecological impact might help designate policies aimed at preventing further introductions and control the invasive populations of already established species.

## Introduction

An increasing body of literature demonstrates the invasive potential of species that have been introduced outside their native range by the direct or indirect intervention of humans, species known as “alien species” (Williamson 1996; Wittenberg and Cock 2001; Pimentel 2002; DAISIE 2009; Kirkendall and Faccoli 2010). In Europe, much interest has been directed to the vectors and pathways of alien species and to the impacts they exert (Nentwig et al. 2010). The knowledge of the invasion processes is truly needed for management purposes of invasive species (Kenis et al. 2007) as well as for conservation issues. Projects such as DAISIE (Delivering Alien Invasive Species Inventories for Europe) have been promoted to investigate European “allodiversity” (i.e., the alien species present in a specific area; Barthlott et al. 1999), and strategies purposed to face biological invasions have been proposed (Genovesi et al. 2004).

It has been shown that over 90% of alien terrestrial invertebrates in Europe are arthropods, with the large majority being insects (Roques et al. 2009). This dominance is due to the high species richness of the class Insecta, as well as to the numerical abundance of individuals, the small body size, and some characteristics of their biology, including short generation time, flexible life-cycle patterns, and feeding habits, combined with their wide adaptability and strong association with humans. Many alien insect species (hereafter referred to as AINS) directly damage human activities (e.g.. agriculture and horticulture) and affect human and animal health (Boettner et al. 2000; NRC 2002; Jenkins 2003; Snyder and Evans 2006; Jensen et al. 2005). It has been also acknowledged that numerous AINS can disrupt the structure and functioning of ecosystems andlargely contribute to biodiversity loss worldwide (Kenis et al. 2009; Kenis and Branco 2010).

The study of invasion processes in arthropods is generally an *a posteriori* study. The early introduction phase is often undetectable, and as a consequence insects become visible only after their stabilization and spread, and when damages they cause are evident. Management efforts, often directed to control already established populations, should be primarily focused on early detection and eradication at the first steps of the invasion process (Kenis et al. 2007). The knowledge of actual allodiversity is useful to promptly address the future. Predictions about alien insect diffusion can be made by assessing, for example, which taxonomic or ecological group is a better invader for that specific area.

Despite the negative impact they exert on ecology and socioeconomy, the risks posed by AINS are still understudied (Kenis et al. 2009) when compared to other taxa such as plants, vertebrates, and aquatic species (Parker et al. 1999; Levine et al. 2003; Long 2003; Kenis et al. 2009). Studies on alien entomofauna are also biased towards economic pests, phytosanitary threats, and vectors of diseases, whereas the ecological impacts of numerous AINS has been only rarely assessed (Kenis et al. 2009).

Roques et al. (2009) reported 1306 AINS in Europe, and showed that their rate of invasion differed among European countries. According to Zapparoli (2008), Italy hosts a total of 728 AINS, which makes the Italian alien entomofauna the richest in Europe. These figures are however an underestimate due to the incompleteness of the collected data (see Audisio et al. 2009) and the bias towards some taxonomic groups (e.g., Pellizzari et al. 2005; Jucker et al. 2009). Inventories should be kept constantly updated and their taxonomic coverage should be as wide as possible in order to forecast both the species that are most likely candidates of invasion and the more vulnerable ecosystems or habitats (Mondor et al. 2007).

**Figure S1.**
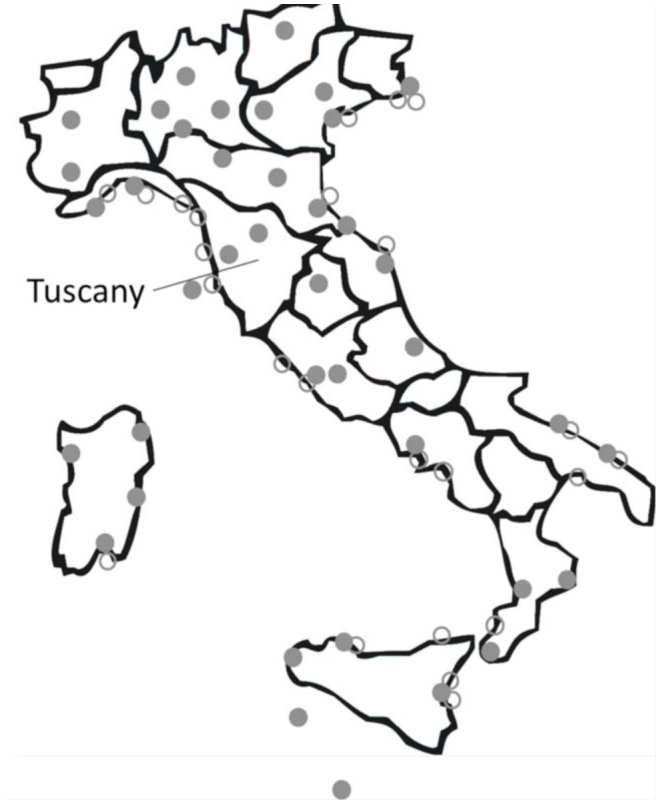
Map of Italy with the main airports and ports, respectively, filled and empty dots. High quality figures are available online.

To fill this gap in knowledge, the status of the alien entomofauna in Italy was updated, a list of AINS in Tuscany (Central Italy) was drawn, and these data were compared with the European inventory of AINS (Roques 2010). Tuscany counts 3,749,074 inhabitants (∼6.2% of the Italian population), on a surface of 22,933 km^2^ (∼7.6% of the Italian surface), with a density of 163.1 inhabitants/km^2^ (compared to 201.2 inhabitants/km^2^ in Italy) (www.istat.it, updated to March 2010). Tuscany (islands included) was selected as a case study because it is one of the best studied region in Italy for entomofauna (Ruffo et al. 2007) and hosts many potential hubs to and from which AINS may be introduced and dispersed, such as two international airports (Pisa and Florence), some important ports (such as Leghorn, Piombino, and Carrara; see Figure S1), and many agricultural and commercial activities (e.g., 7240 ha of nurseries, 31% of the regional surface, with 1767 nurseries in Pistoia, 5.5% of the total number of these activities in Italy; Regione Toscana - Settore Sistema Statistico Regionale 2006).

## Materials and Methods

Alien species were defined as species introduced, after 1492 A.D., outside their natural past or present distribution, according to C.O.P. (2002) and Zapparoli (2008). Parautocthonous species (i.e., established species introduced before 1492 A.D.) and species subject to translocation (i.e., subject to movement from one region to another within the same country) were not included in the analysis.

Data were obtained from over 300 papers published in scientific and “gray” literature, from direct information by specialists, and from recent field research by the authors. Data mainly come from Italian checklists (Minelli et al. 1993–1995; Ruffo et al. 2007) and from reviews on specific groups (e.g., Pellizzari et al. 1997; Nicoli Aldini 2003; Ratti 2007; Abbazzi et al. 2009; Jucker et al. 2009). For each species (updated to November 2011), the following information was collected: 1) biology (feeding habits and reproductive strategies), 2) native distribution (zoogeographic regions: Palearctic West and East, Nearctic, Afrotropic, Neotropic, Oriental and Australasiatic (Zapparoli 2008)), 3) status (whether established, i.e., naturalized or acclimatized, or not, i.e., intercepted, extinct, or eradicated), 4) mode of arrival (intentional/unintentional), 5) pathway of introduction (biocontrol, if released as biological control; ornamental, if imported in association with ornamental plant species; culture, if imported in association with agriculture; trade, if introduced due to other commercial activities and transport network), and 6) date of first introduction in Italy and in Tuscany, when available. Date of first introduction refers to either the exact or the approximate year reported in literature or, when this not available, to the year of publication of the first record. When no documentation was available for a given category, or the information was dubious or anecdotal, ‘unknown’ was recorded. The Checklist of the Italian Fauna compiled by Stoch (2003) was used to assess the relative abundance of AINS in Tuscany across orders with respect to the Italian indigenous entomofauna.

**Figure 1.**
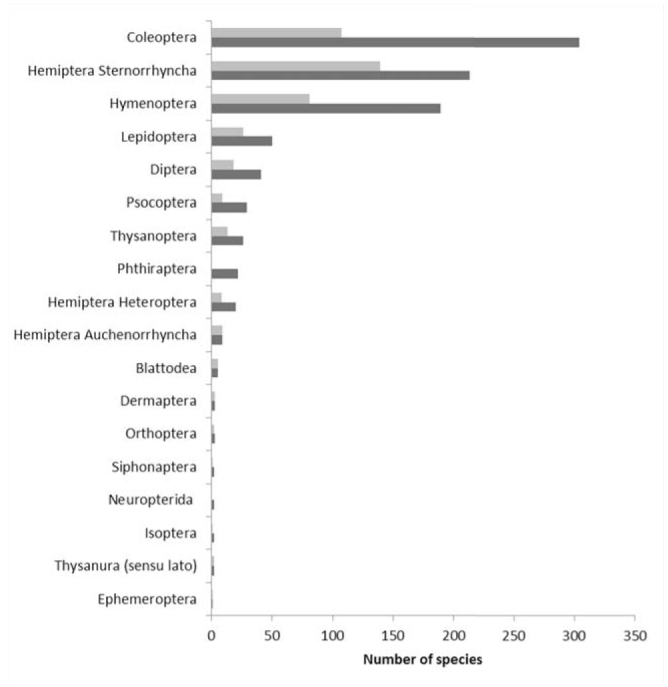
Total (dark grey) and established (light grey) alien insect species in Italy. Total alien insect species: n = 923; established alien insect species: n = 425. High quality figures are available online.

The information about the abundance of AINS (number of species and relative frequency in percentage for each order, suborder, or group of orders) were analyzed at three geographical scales: local, national, and continental (Tusca-ny, Italy, and Europe). For Italy and Tuscany, both the total AINS and the established AINS were analyzed, whereas for Europe only the established species “alien to Europe” *sensu* Roques (2010). The term “established” was used to indicate naturalized and acclimatized species *sensu* Pyšek et al. (2009). The worldwide distribution of insects, as reported by Grimaldi et al. (2005), was used to understand whether the distribution of AINS across orders in Europe and Italy reflects their world's distribution. Temporal trends of introduction on 10-year intervals (starting from 1950) and Zoogeographic regions of origin were also reported.

Pearson's linear regression (r^2^) was used to analyze temporal trends of cumulative introduction. Differences between relative frequencies (%) were computed to compare data of four geographical scales: Tuscany, Italy, Europe, and the world. Ratios (%) were computed between the AINS (total AINS for Italy and established AINS for Europe) and the total number of insect species in the world across orders and between the established AINS and the total AINS in Italy. Statistical comparisons among frequency data were made using Wilks' test after Williams' correction (statistic: G). The level of significance at which the null hypothesis was rejected was α = 0.05.

## Results

**Figure 2.**
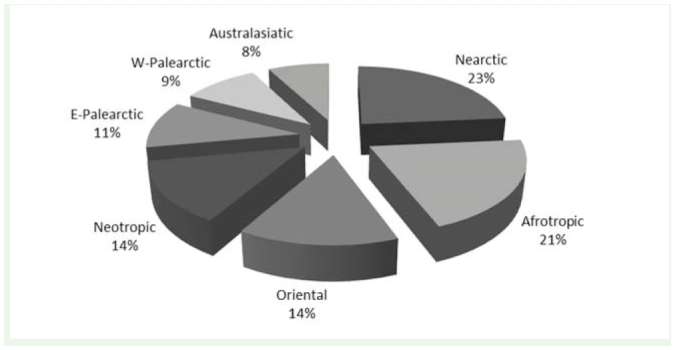
Native regions of alien insect species in Italy (n = 687). Cryptogenic (n = 108) and pantropical (n = 47) species are not shown. High quality figures are available online.

**Figure 3.**
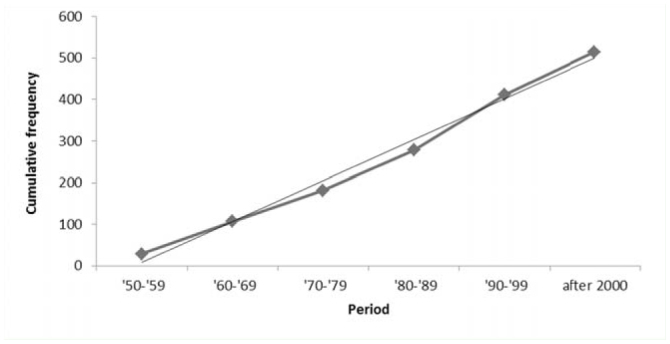
Linear regression between the cumulative number of recorded alien insect species in Italy and time. High quality figures are available online.

The number of AINS in Italy amounted to 923, including 425 (46%) established species. The abundances of total and established AINS across orders are shown in Figure 1. Species mainly come from the Nearctic (23%) and Afrotropic Zoogeographic regions (21%) (Figure 2). Their rate of introduction is described by a linear regression model (r^2^ = 0.989; df = 1; t = 18.813; *p* < 0.001; Figure 3).

**Figure 4.**
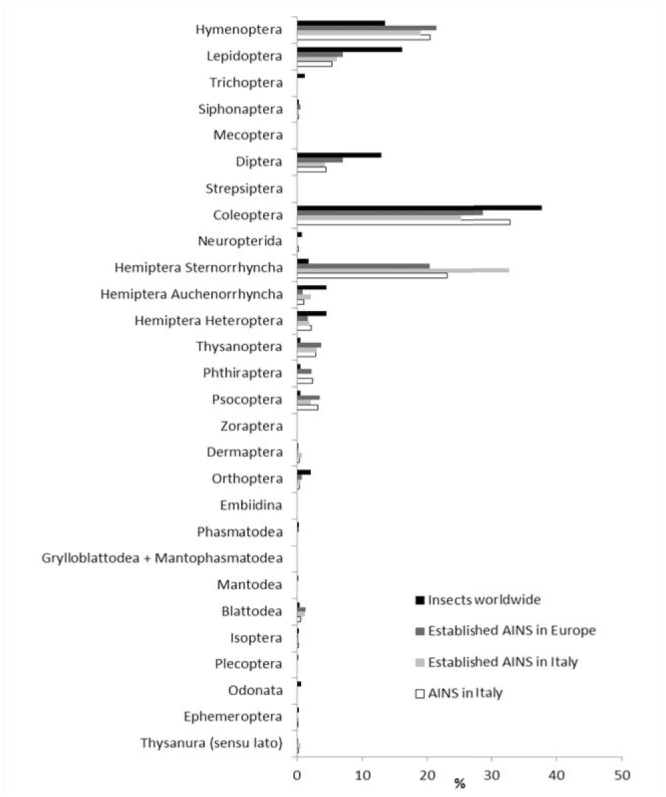
Proportion of each order of insects in the world, established alien insect species in Europe, established alien insect species in Italy, and total alien insect species in Italy. High quality figures are available online.

Hymenoptera and Hemiptera (Sternorrhyncha) included more AINS than expected from their worldwide distribution, whereas Lepidoptera, Diptera, and Coleoptera comprised less AINS than expected (Figure 4). The same pattern was found in Europe.

Italy and Europe also showed a similar ratio across orders between the number of AINS (the total number for Italy and the number of established AINS for Europe) and the total number of insect species in the world (Figure 4). The highest percentages were found for Hemiptera (Sternorrhyncha), Thysanoptera, Phthiraptera, and Psocoptera.

**Figure 5.**
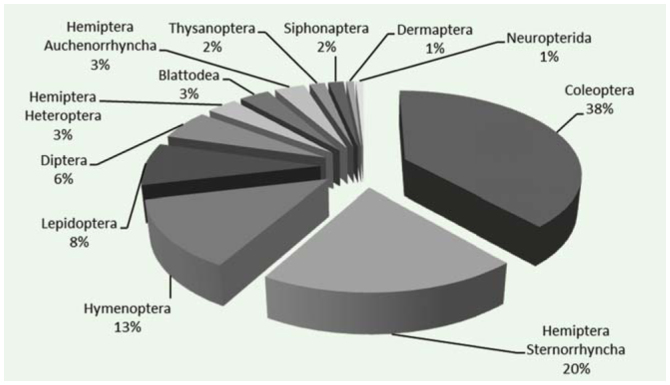
Percentage of alien insect species across orders in Tuscany (n = 122). High quality figures are available online.

AINS in Tuscany reached a total of 122 species (Table 1) belonging to 10 orders, mainly Coleoptera (38%), Hemiptera (Sternorrhyncha and Auchenorrhyncha) (23%), and Hymenoptera (13%) (Figure 5). Ninety-two percent of species had established populations. Twelve percent of species had been recorded only in Tuscany across Italy. Tuscany hosted 26% of established AINS in Italy.

**Figure 6.**
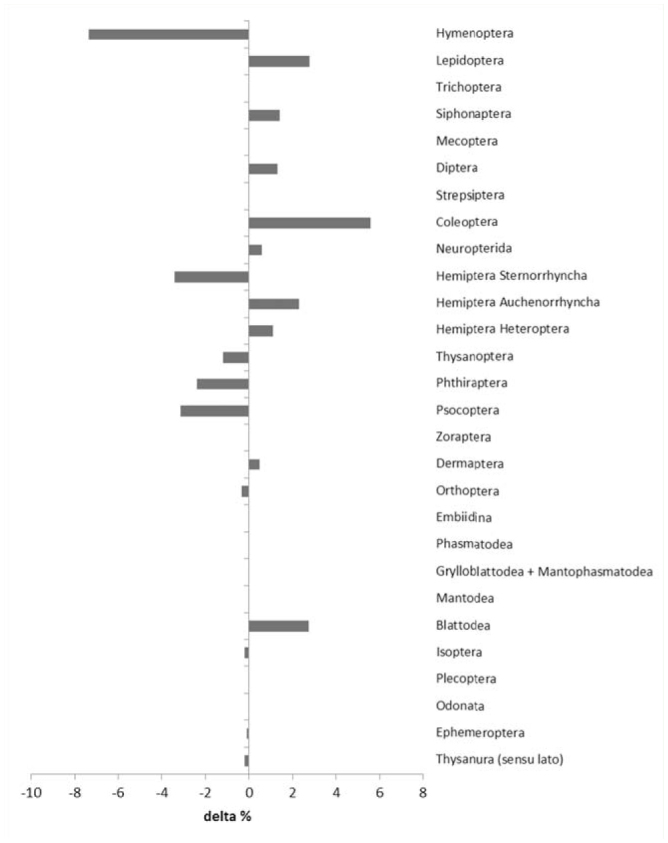
Difference (indicated with delta) in % between the relative frequency of total alien insect species in Tuscany and the relative frequency of total alien insect species in Italy across orders. High quality figures are available online.

**Figure 7.**
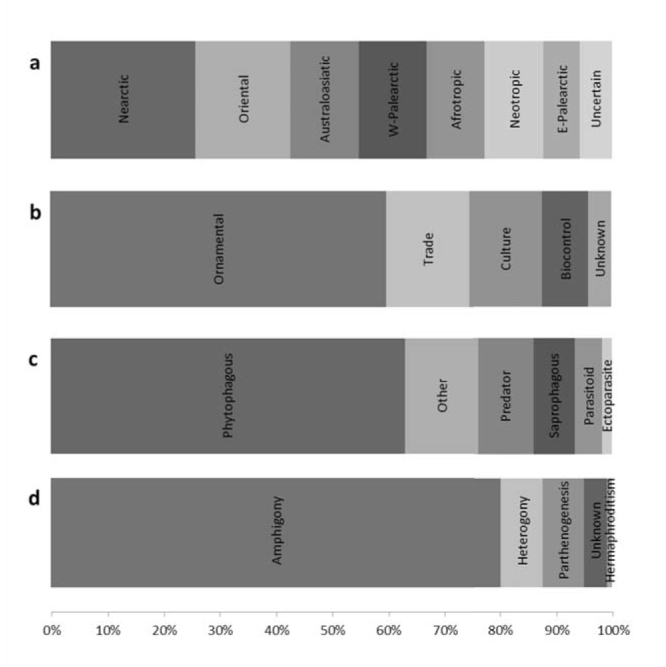
a: Native Zoogeographic regions (%) (cryptogenic species are under the label “uncertain”); b: pathways of introduction (%); c: feeding habits (%); and d: reproductive strategies (%) of the total alien insect species recorded in Tuscany (n = 122). High quality figures are available online.

Alien Coleoptera and Lepidoptera were more abundant in Tuscany than in Italy, whereas alien Hymenoptera and Hemiptera (Sternorrhyncha) were less frequent (Figure 6). Nearctic species represented the largest group (26%) of aliens (Figure 7a).

The majority of introductions were accidental (intentional: 9; unintentional: 113; G = 104.46; df = 1; *p* < 0.001), with species mostly associated with vegetable items (Figure 7b). AINS were mainly phytophagous (63%) (Figure 7c) and amphigonic (80%) (Figure 7d).

## Discussion

Our study confirmed that Italy was both one of the most invaded European country by AINS and one of the best studied from the invasion biology perspective (Roques 2010). A relatively high rate of introduction of insects to Tuscany was also shown, with a consequent abundant entomological allodiversity.

In Europe, as a probable effect of both globalization and more in-depth studies, the trend of introduction of arthropods is increasing, with an average of 19.6 new alien species per year between 2000 and 2008 (Roques 2010). This rate can be considered informative for insects because Roques (2010) found that insects amount to 86% of total arthropods alien to Europe. In Italy, a constant linear trend of new insect species introduction (about 10 per year) was found since the Second World War. The dates of first introduction/record of AINS in Tuscany are not available for many species, especially if the species was first seen in a different region of Italy, so a computation of the regional rate of introduction was unreliable.

As expected from the current trading routes (Phytosanitary Service of Tuscany Region, personal communication), North America (Nearctic region) and Asia (Oriental, Australasiatic, and East Palearctic regions) are the most frequent donor regions of AINS for Italy. Surprisingly, many species come from the Afrotropic Zoogeographic region, probably due to its geographical closeness to Italy and the possibly less effective controls in the countries of origin.

In both Europe and Italy, alien Hemiptera (Sternorrhyncha) are more frequent than expected because they are skilled invaders (Pellizzari and Germain 2010). Hemiptera (Sternorrhyncha) have the highest number of established species (as reported in Pellizzari et al. 1997; Zapparoli 2008), possibly due to their small size and ability to travel on plant material, but also due to their biology such as parthenogenesis (quite common in the order), high fecundity (Pellizzari and Germain 2010), and short generation time, which are all traits that favor their quick colonization.

In Italy, a small proportion of established AINS were found in the orders Hymenoptera and Coleoptera, as compared to Hemiptera (Sternorrhyncha). Most alien Hymenoptera are parasitoids or hyperparasitoids. They were intentionally introduced for biological control in the “golden years” of biocontrol efforts, between 1950 and 1999 (Rasplus et al. 2010), but may not have established reproductive populations. Coleoptera is the richest order of insects, with a broad range of feeding and reproductive strategies leading to a variable success of colonization across families and genera. For instance, Cerambycidae includes *Anoplophora chinensis* Forster, which damages a wide range of broadleaved trees and shrubs, and *Neoclytus caprea* (Say), which was intercepted in imported timbers but never established (Ratti 2007).

Moreover, the relatively small size of Phthiraptera, Psocoptera, and Thysanoptera, along with their cryptic habits and association with animals and with stored products, make them successful invaders (Kenis and Roques 2010; Reynaud 2010; Schneider 2010). Two paradigmatic cases are Blattodea (strictly anthropophilic) and Hemiptera (Auchenorrhyncha) (mostly Cicadellidae feeding on host plants), insects that quickly became established after introduction. Alien Lepidoptera, Diptera, and Coleoptera are less frequently found than expected given their worldwide abundance. In Italy, the frequency of established Lepidoptera is similar to Europe as a whole, suggesting that that butterflies and moths have a lower introduction rate than expected given their worldwide abundances. The low frequency found for Lepidoptera, which have good dispersal capability, can be explained by their sensitivity to ecological barriers such as the absence of host plants or climate matching (Devictor et al. 2012). Lepidoptera are well-studied across Italy and Europe (L. Dapporto, Department of Biology, University of Florence, personal communication), as are other taxa of economic interest, such as Hemiptera Sternorrhyncha (Pellizzari et al. 1997). On the contrary, there is a paucity of studies for some groups among Diptera and Coleoptera (e.g., Cucujoidea), which can explain the under-representation for these orders.

Tuscany showed the same pattern as Italy as a whole for the relative abundance across orders of AINS and the areas of origin of AINS, except for Lepidoptera and Coleoptera, who appeared to be more abundant possibly because they are more traditionally more studied in Tuscany than in the rest of Italy (Sforzi et al. 2001).

Even though entomological research in Tuscany is intense, and Tuscany is one of the few Italian regions with a “Red List” of insects (Sforzi et al. 2001), data about AINS are scarce or difficult to retrieve, especially forspecies without any impact on the economy or human health. An emblematic case of the limited knowledge of AINS is *Stenopelmus rufinasus* Gyllenhal (Abbazzi et al. 2009), a species introduced into several countries for the biological control of *Azolla* sp. (e.g., McConnachie et al. 2004; Nelson 2007). Although the native range of this frond-feeding weevil is North America, it is classified as protected in Tuscany (L.R. 56/2000) (Abbazzi et al. 2001). In Italy, its introduction was not intentional and, its presence in the wild is not preserved, due to its positive effects on ecosystems, or supported by reintroduction for biological control. After our report, the species has been removed from the Tuscan list of species of particular concern (Repertorio Naturalistico Toscano; Bartolozzi and Cianferoni 2012), but it should also be removed from regional regulations.

Our study showed that Tuscany, with a total of 122 AINS, should be regarded as a hotspot of entomological allodiversity (13% of the total AINS in Italy), as also confirmed by the high fraction (12%) of AINS still confined in this region. This high allodiversity might be due to the role of airports and ports, along with the intense trade of ornamental plants, all of which can serve as gateways for AINS introductions. AINS in Tuscany are mainly phytophagous and amphigonic, and were accidentally introduced in association with vegetable items as ornamental plants or crops. For example, the red palm weevil, *Rhynchophorus ferrugineus* (Olivier), one of the worst pest of palms that is rapidly spreading in central and southern Italy, was first recorded in Italy from a nursery in Pistoia in 2004 (Sacchetti et al. 2005, 2006; Longo et al. 2008). Tuscany also offers a wide variety of habitats and ecological niches (Sforzi et al. 2001; De Dominicis et al. 2010) that may fa-vor both the settlement and the spread of AINS.

Nineteen species (16% of the AINS in Tuscany) are known to be invasive, being included in international databases on invasive alien species such as DAISIE (www.europe-aliens.org) and GISD (Global Invasive Species Database by Invasive Species Specialists Group; www.issg.org), or mentioned in EPPO Quarantine and Alert lists (www.eppo.org; each list was accessed 8 August 2011). In particular, 15 species (12% of the AINS in Tuscany) are of phytosanitary interest, their introduction, spread, and impact being investigated by the National Plant Protection Organization. In Tuscany, 23% of the species listed in EPPO A2 and Alert lists (n = 66) are present.

For the species that are not included in the EPPO list, there is no legal instrument banning their introduction, except for some species that affect human and animal health, such as *Aedes albopictus* (Skuse). The International Health Regulation administered by the World Health Organization indicates measures on prevention and control of harmful species, but these measures are directed to contain outbreaks of new diseases without monitoring trade and travel-related introductions of the vectors of the diseases (Keller et al. 2010).

Certainly, the total AINS recorded in Tuscany, as well as that recorded in Italy as a whole, is an underestimation of insect allodiversity. Notwithstanding the increased scientific interest for biological invasions in the latest 50 years, there is still a gap in knowledge about many insect taxa and some functional groups. Despite this limitation, our study is useful in identifying important pathways of introduction. In particular, ornamental plants are the most important vectors for the introduction of AINS into Tuscany, as well as the rest of Italy and Europe (Kenis et al. 2007). Our study also indicates that, for almost all the recorded species, the impacts exerted on the recipient communities and ecosystems, as well as the distribution at local level, are still unknown. Further studies are thus needed to investigate these issues, with the purpose of designating policies that might prevent further introductions and control the invasive populations of already established species. Finally, we believe that it is highly important and desirable that the alien insects list we have drawn for Tuscany will be constantly updated in future. This upgraded database should better address the management efforts towards early detection and eradication at the first steps of the insect invasion process. Moreover, we hope that our work could be a basis to stimulate the drawing of similar entomological allodiversity databases at local levels. The knowledge of the allodiversity of neighboring regions is essential to prevent and better manage the arrival of new alien species.

**Table 1.**
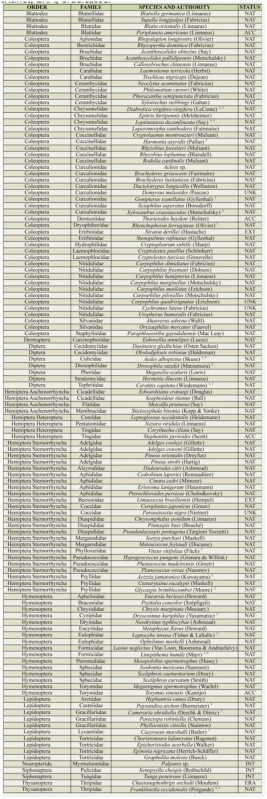
Alien insect species recorded in Tuscany. Roques et al. (2010a, b) was mainly followed for species' nomenclature. The species, alphabetically ordered, are listed in: 1 EPPO A2 quarantine list, ^2^ EPPO Alert list; ^3^ 100 of the worst invasive alien species from DAISIE; ^4^ 100 of the worst invasive alien species from GISD. Abbreviations for status are as follow: NAT, naturalized; ACC, acclimatized; INT, intercepted; EXT, extinct; ERA, eradicated.
